# Study protocol of a randomized controlled superiority trial of Huddinge Online Prolonged Exposure therapy (HOPE) for adults with posttraumatic stress disorder

**DOI:** 10.1186/s13063-025-09361-0

**Published:** 2025-12-12

**Authors:** Maria Bragesjö, Volen Z. Ivanov, Erik Andersson, Christian Rück

**Affiliations:** 1 Centre for Psychiatry Research, Department of Clinical Neuroscience, Karolinska Institutet & Stockholm Health Care Services, Region Stockholm, Stockholm, Sweden; 2 Department of Psychology, Department of Clinical Neuroscience, Karolinska Institutet, Stockholm, Sweden

**Keywords:** Posttraumatic stress disorder, Trauma-focused cognitive behavior therapy, Digital treatment, Prolonged exposure, Therapist-assisted, Therapist-guided

## Abstract

**Background:**

Cognitive behavioral therapy (CBT) is considered a first-line treatment for posttraumatic stress disorder (PTSD) but is rarely available in regular care. Action is clearly needed to increase treatment availability. A possible solution to improve access to treatment would be the use of remotely delivered digital intervention. Internet-delivered CBT may also carry several other advantages compared to traditional psychological treatments (e.g., less therapist time per patient, bridging geographical distances between therapist and patient, and provision of a standardized intervention). In a pilot study conducted within a psychiatric outpatient setting, a digital therapist-guided prolonged exposure program (Huddinge Online Prolonged Exposure; HOPE) demonstrated preliminary effectiveness, patient acceptability, and feasibility. This project will build upon these findings and evaluate whether HOPE is effective in reducing PTSD symptoms.

**Method:**

The study is a randomized controlled superiority trial, with assessors masked to treatment allocation. Two hundred eighty-six participants will be randomly allocated to receive either 10 weeks of HOPE or an active control condition (therapist-guided internet-delivered psychoeducation, relaxation, and support). The primary outcome is the severity of symptoms of PTSD for the last month measured by the clinician-administered PTSD scale for DSM-5 (CAPS-5). Secondary outcomes include self-rated measures of symptoms of PTSD and complex PTSD, depression, quality of life, cost effectiveness, mediators of change, dropout rates, and adverse events.

**Discussion:**

This study will be the first to compare digital prolonged exposure (HOPE) with an active control condition and not exclude patients with severe or complex PTSD. The strength of the study is that it is conducted in a clinical setting with wide eligibility criteria and the use of gold-standard measures to assess outcomes. Potential challenges in the execution of the trial include participant recruitment, retention, and adherence to treatment and therapist retention.

**Trial registration number:**

Clinicaltrials.gov; NCT05934162. Registered 6th July 2023. Open Science Framework https://osf.io/dsg9p/. Registered 2nd September 2023.

## Background {6a}

Posttraumatic stress disorder (PTSD), as defined in the Diagnostic and Statistical Manual of Mental Disorders, Fifth Edition, is a debilitating psychiatric condition characterized by distressing symptoms such as intrusions of the traumatic event, inability to be around trauma reminders, cognitive and mood changes, and arousal symptoms and is associated with considerable functional impairment and comorbidity [1]. PTSD is one of the most common psychiatric disorders, with an estimated lifetime prevalence of approximately 5.6% [2] If left untreated, extensive data show a significantly increased risk for substance use, suicidality, and poorer mental health/functioning [3, 4] as well as impaired physical health [5].

Individual trauma-focused CBT, such as prolonged exposure (PE), is considered a first-line treatment for PTSD is recommended as a first-line treatment for adult PTSD across major guidelines, including NICE, the 2023 VA/DoD guideline, and the APA clinical practice guideline but is seldom available in regular care [6]. Given the large group of afflicted and the treatment gap, action is clearly needed to increase availability. A possible solution to improve access to evidence-based treatment would be the use of remotely delivered digital treatment. Internet-delivered CBT may also carry several other advantages compared to traditional psychological treatments (e.g., less therapist time per patient, bridging geographical distances between therapist and patient, and provision of a standardized intervention). Recently, the NICE Early Value Assessment recommended two digital treatment programs for adults with mild to moderate PTSD based on the results of two large randomized controlled trials, for use in the NHS while further evidence is generated. The first study (RAPID, *n* = 196) showed that internet-delivered therapist-guided CBT was noninferior to face-to-face CBT treatment at 16 weeks, with improvements maintained to 52 weeks [7]. The other study (STOP-PTSD, *n* = 217) demonstrated that internet-based therapist-guided cognitive therapy for PTSD was superior to internet-based stress management therapy at 13 weeks, with outcomes and cost-effectiveness assessed through 26 and 39 weeks [8]. PE has thus far been evaluated in a digital format in two previous settings, among military personnel and veterans in the USA (Web-PE; [9]) and for recently traumatized individuals in the general Swedish population (Condensed internet-delivered prolonged exposure CIPE; [10, 11]).

This project is the first large RCT to evaluate therapist-guided digital PE for PTSD and not exclude severe cases of PTSD and complex PTSD as often seen in psychiatric settings.

## Objectives {7}

The main objective of the trial is to test whether our newly developed digital treatment (Huddinge Online Prolonged Exposure; HOPE) is more effective than the active comparator in reducing PTSD symptoms. The trial will also include a health-economic evaluation from both the treatment provider perspective (i.e., direct costs such as costs for healthcare personnel) and a broader societal perspective (i.e., indirect costs such as sick leave). We will also investigate processes of change during treatment and predictors of treatment outcome.

## Trial design {8}

**Fig. 1:**
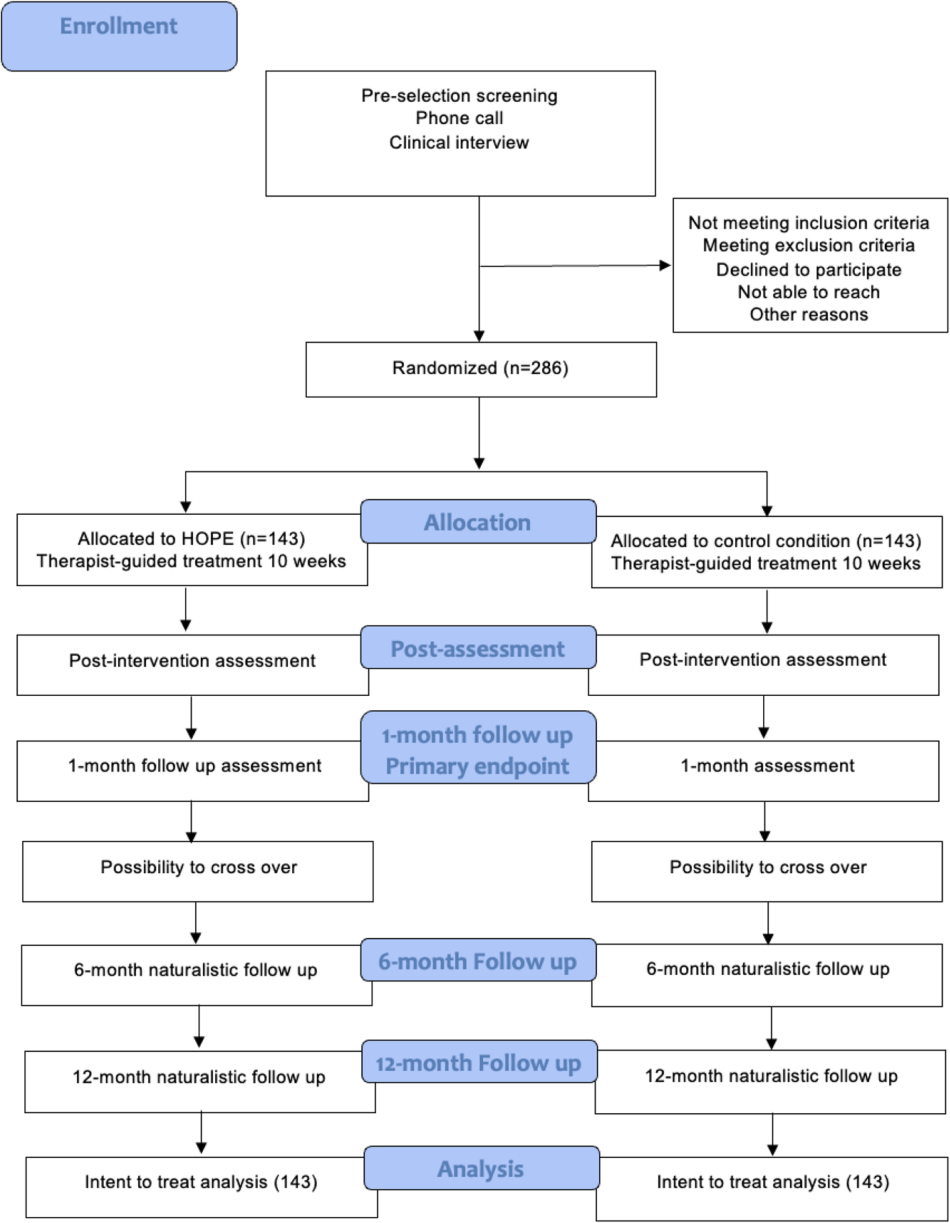
CONSORT flowchart

The trial uses a single-blind (blinded assessors), randomized (1:1), controlled, parallel-group, superiority design comparing 10 weeks of either therapist-guided HOPE to an active control condition for adults with PTSD residing in Sweden. We aim to recruit 286 participants who will be assessed pretreatment, during treatment, posttreatment, and 1 month after treatment completion (primary endpoint). Naturalistic follow-up will be conducted at 6- and 12-month follow-ups. The Consolidated Standards of Reporting Trials (CONSORT) flow chart of the trial is shown in Fig. 1. The data analytic plan was published on the Open Science Framework (OSF) before data collection began.

The research study received ethical approval from the Swedish Ethical Review Authority under ID 2023-02329-01 and was preregistered on both Clinicaltrials.gov and OSF before the commencement of data collection. The reporting of data will adhere to the guidelines set forth in The Consolidated Standards of Reporting Trials statement for nonpharmacological trials (CONSORT) and the Consolidated Health Economic Evaluation Reporting Standards (CHEERS) regardless of the study’s outcome. The trial will adhere to the principles of Good Clinical Practice (GCP).

## Methods

### Study setting {9}

The trial will take place in a publicly funded outpatient psychiatric clinic in Stockholm, Sweden. Self-referral is possible through a dedicated secure study site.

### Eligibility criteria {10}

We will employ broad eligibility criteria to maximize the external validity of the study. Participants with comorbid conditions, such as neuropsychiatric disorders, depression, and substance abuse, will be considered for inclusion provided they meet all other inclusion/exclusion criteria, with PTSD being their primary diagnosis. Eligibility is determined via a two-stage process: (1) a participant-completed web-based screening battery for common mental disorders (including PTSD symptoms, depression/anxiety, alcohol/drug use, psychosis/mania, and suicidal ideation), followed by (2) a clinician-administered diagnostic assessment to confirm PTSD and evaluate risk.

Please see Table 1 for the full inclusion and exclusion criteria.

**Table 1:** Inclusion and exclusion criteria

**Inclusion criteria**	≥ 18 years of age
	Primary diagnosis of PTSD according to the Diagnostic and Statistical Manual of Mental Disorders, fifth edition (DSM-5)
	Signed informed consent
	Have had a stable dose of any psychotropic medication for at least 4 weeks prior to study entry
	Being fluent in Swedish
	Daily access to a computer or device with internet connection
**Exclusion criteria**	PTSD is not the primary concern
	Serious mental health symptoms, such as mania, psychosis, alcohol, or substance use disorders or current suicide risk warranting immediate clinical attention
	Ongoing trauma-focused CBT or Eye movement desensitization and reprocessing therapy
	Ongoing trauma-related threat (e.g., living with a violent spouse)

### Informed consent {26a}

During the self-referral process, informed consent will be obtained through a secure study platform utilizing a two-step authentication system.

### Additional consent provisions for collection and use of participant data and biological specimens {26b}

This trial does not involve collecting biological specimens for storage.

## Interventions

### Explanation for the choice of comparators {6b}

The trial will compare therapist-guided digital prolonged exposure (HOPE) to a digital therapist-guided active control condition to assess its efficacy. A matched digital comparator preserves parity in modality, platform, therapist contact, and expectancy, enabling estimation of the incremental benefits and potential adverse effects attributable to trauma-focused exposure. The comparator includes psychoeducation, stress-management skills, and supportive therapist contact, and explicitly excludes imaginal and in vivo exposure and other trauma-focused techniques. After the 1-month post-treatment follow-up (primary endpoint), participants may access the alternative condition without therapist guidance. This timing preserves the integrity of the primary endpoint while improving ethical access.

### Intervention description {11a}

Prolonged exposure is considered one of the first-line treatments for PTSD and is recommended in available treatment guidelines [6]. In this trial, PE is delivered digitally as HOPE, a therapist-guided, internet-based program over 10 weeks comprising six sequential modules [11]. Modules are content packages rather than single “sessions”: participants work with each module across several days or weeks, completing multiple practice exercises within and between modules. The treatment mirrors face-to-face PE in structure and components, psychoeducation, in vivo exposure, imaginal exposure with processing, and relapse-prevention planning and progression is driven by practice completion and clinical milestones.

## Comparator

Active control will use established CBT interventions such as psychoeducation about trauma and PTSD, relaxation, stress management techniques, and controlled breathing but will not include any exposure. The treatment will be given in a digital format with therapist support and consist of six sequential treatment modules that the participants are expected to complete in 10 weeks.

## The role of the therapist

Each participant is assigned a designated therapist who provides guidance and support in both trial arms, answers questions, and gives feedback on digital worksheets. To ensure consistent access, therapists log into the platform on three pre-specified weekdays (Monday, Wednesday, and Friday) for all participants to review progress and to address messages even if no new message has been posted. Guidance is proactive: each participant receives at least one substantive feedback message per week irrespective of participant-initiated contact, and therapists reply within 48 h on business days to any incoming message. Completion of a module and submission of exposure worksheets trigger targeted feedback within 48 business hours. This login cadence and response-time standard are identical across arms and are communicated to participants at onboarding. Brief telephone check-ins may be conducted when clinically indicated; such contacts are considered protocol deviations and are logged in the eCRF.

Participants complete the PCL-5 weekly within the platform, accompanied by an open-ended question about any adverse events including perceived intensity and duration. When PCL-5 scores or AE reports indicate exacerbation, the default response is delivered via secure platform messaging: the therapist validates and normalizes transient symptom increases, screens for risk, reviews adherence and possible safety behaviors, and titrates exposure intensity (e.g., temporarily shortening imaginal exposure assignments or stepping down in vivo tasks, then re-escalating as tolerated) while maintaining fidelity to PE. If elevated distress persists or risk is suspected, the therapist may conduct a brief telephone check-in to complete a focused risk assessment and agree on next steps; such calls are treated as protocol deviations and logged in the eCRF. All routine assessments and contacts are remote, and no face-to-face visits are planned. Acute risk is managed per the study safety protocol, including immediate clinical escalation and referral to regular care services (e.g., emergency psychiatric services, local outpatient psychiatry, or the emergency department), with activation of emergency services when indicated; all actions are documented in the eCRF.

### Criteria for discontinuing or modifying allocated interventions {11b}

Participants may withdraw their consent to participate in the treatment and the trial at any timepoint. Suicidal ideation triggers a structured suicide-risk assessment (e.g., C-SSRS plus focused interview on intent, plan, means, and protective factors) and, if indicated, referral. All safety assessments are conducted remotely (telephone or secure video), no face-to-face visits are planned. The intervention may be temporarily titrated or discontinued for (a) acute suicide risk requiring urgent care or (b) emergent medical/psychiatric conditions that render participation unsafe. Acute risk is managed per the study safety protocol with immediate escalation and referral to regular care (e.g., emergency psychiatric services, local outpatient psychiatry, emergency department). Participants who discontinue the intervention are invited to continue all scheduled outcome assessments when clinically appropriate.

### Strategies to improve adherence to interventions {11c}

The platform sends automated SMS reminders at each scheduled weekly monitoring point and an SMS notification whenever a therapist posts a message. If a participant has not logged in for 3 consecutive days, the therapist sends a personalized SMS via the platform to prompt engagement. If inactivity reaches 5 consecutive days, the therapist attempts telephone contact; if phone contact cannot be established, a letter is mailed within the same week. Automated digital reminders continue, and the therapist re-attempts contact weekly thereafter unless the participant withdraws consent. (This escalation schedule is identical across trial arms.)

Participants who discontinue treatment are retained in follow-up whenever possible and invited to complete all scheduled outcome assessments; this approach minimizes attrition bias and supports valid intention-to-treat analyses.

The therapist delivering the treatments in the trial will comprise resident psychologists (a 1-year post-degree licensure period following a 5-year MSc in Psychology). All clinicians are based at a specialized PTSD unit and deliver treatment in both trial arms. All therapists will receive training in and a written treatment manual for both treatment arms. Supervision by certified supervisors and trainers in prolonged exposure and/or CBT will be offered weekly.

Blinded assessors will undergo a minimum of a full-day training in the CAPS-5 prior to conducting clinician-rated assessment with CAPS-5 and practice by rating videos of fictious PTSD case examples. Assessors who deviate more than 10% of the CAPS-5 total score agreed upon by one of the authors (MB) and score more than one point differently on a maximum of four single items will need to retake the training. The assessors are offered weekly supervision.

### Relevant concomitant care permitted or prohibited during the trial {11d}

As per the inclusion and exclusion criteria, the participant must have had a stable dose of any psychotropic medication for at least 4 weeks prior to study entry, and no other ongoing trauma-focused CBT or eye movement desensitization and reprocessing therapy is permitted during the trial. Data on any potential alterations to medication or the initiation of trauma-focused CBT or Eye Movement Desensitization and Reprocessing therapy will be collected during the follow-up assessment.

### Provisions for posttrial care {30}

At the end of the study, participants assessed as in need of further treatment will be helped in finding other adequate treatments.

### Outcomes {12}

The primary outcome measure is the CAPS-5, the gold-standard clinical interview that evaluates the severity of PTSD symptoms and determines the presence of PTSD in the past month [12]. This evaluation will be conducted at multiple time points: upon enrollment, 1 month after completing the treatment (primary endpoint), and during the 6- and 12-month follow-up assessments. The CAPS-5 total severity score has been shown to exhibit strong internal consistency (Cronbach’s *α* = 0.88) and interrater reliability (intraclass correlation coefficient = 0.91), along with favorable test–retest reliability (intraclass correlation coefficient = 0.78) [12]. Treatment response will be considered achieved if there is a minimum of 10 points improvement on the CAPS-5 between baseline and the participant’s last available measurement within the baseline to 12-month follow-up period (adapted from [13]). Remission will be defined as the absence of a PTSD diagnosis or a CAPS-5 total symptom severity score below 12 [13]. To be classified as having achieved recovery, participants must maintain their remission status at both the 6-month and 12-month follow-ups.

Secondary self-rated outcome measures include the Posttraumatic Stress Disorder Checklist for DSM-5 (PCL-5) [14] and the International Trauma Questionnaire (ITQ) [15] to capture self-rated symptoms of PTSD and complex PTSD. The Patient Health Questionnaire (PHQ-9; [16]) and the EuroQol 5-dimensions (EQ-5D; [17]) will be used to assess the level of depressive symptoms and quality of life. Treatment response on the PCL-5 will be defined as a drop of 10 points or more. For remission, a score below 30 will be used, which has been identified as indicative of probable PTSD in the Swedish version of the PCL-5 [18].

To calculate quality-adjusted life-years for cost-utility analysis, participants will complete the Assessing Quality of Life 6 Dimensions (AQoL-6D; [19]). Data concerning the utilization of medical resources, medication, social care, absenteeism, and presenteeism in relation to employment will be collected using The Treatment Inventory of Costs in Patients with psychiatric disorders (TIC-P; [20]). Moreover, information on adverse events will be gathered using the Negative Effects Questionnaire (NEQ) immediately after treatment completion and the upcoming assessment points [21]. Participants will also receive an open-ended question asking for the occurrence of adverse events weekly during treatment.

#### Treatment variables

Data will also be collected on the number of completed modules, dropouts of treatment, sent messages, and therapist time spent in the digital platform for each participant and any additional time needed, for example, phone calls outside the platform. A treatment completer is defined as a participant who completed all treatment modules or responded early. Early recovery is defined as the participant showing a positive response after completing at least 3 modules, with a minimum of a 10-point reduction on the PCL-5 or achieving a total sum score below 30, and both the participant and therapist agreeing to conclude the treatment. Participants who discontinue treatment or experience early recovery will be encouraged to continue their participation in the remaining outcome assessment points.

Ratings on the subjective units of distress (SUD) will be collected during exposure exercises (pre, during, post and maximum), and harm expectancies will be assessed using digital worksheets. The Credibility/Expectancy Questionnaire (CEQ; [22]) and Working Alliance Inventory–Short Form Revised (WAI; [23]) will be utilized to assess treatment credibility and working alliance between the treatment arms.

### Participant timeline {13}

The participant timeline and timepoints for assessment for the study can be seen below in Table 2.

**Table 2:** Participant timeline and timepoints for assessment

	Enrollment	Allocation	Baseline	Weeks 1–10	1-months follow up (primary end-point)	6-month follow-up	12-month follow-up
**Enrollment:**							
Eligibility screen	X						
Informed consent	X						
Allocation		X					
Treatment:							
HOPE				X			
Control condition				X			
**Clinical administered instruments:**							
CAPS-5	X				X	X	X
**Self-rated instruments**							
PCL-5			X	X	X	X	X
ITQ			X		X	X	X
PHQ-9			X		X	X	X
EQ-5D			X		X	X	X
AQoL-6D			X		X	X	X
TIC-P			X		X	X	X
NEQ					X	X	X
CEQ*							
WAI-SR**							
SUD***				X			

*Abbreviations: AQoL-6D* Assessing Quality of Life 6 Dimensions, *CAPS-5 *Clinician-Administered PTSD Scale for DSM-5, *CEQ *Credibility/Expectancy Questionnaire, *ITQ *The International Trauma Questionnaire, *NEQ *Negative Effects Questionnaire, *SUD *Subjective Units of Distress Scale, *TIC-P *Treatment Inventory of Costs in Psychiatric Patients, *PHQ-9 *Patient Health Questionnaire, *WAI-SR *Working Alliance Inventory–Short Form Revised

*CEQ is administered after the first module in each treatment format

**WAI-SR is administered at start of the third treatment week in each treatment format

***SUD rating will only be collected in the HOPE group each time digital worksheets for exposure are filled in

Interested applicants can self-refer to the study through a dedicated secure website by completing an online screening battery comprising questions about inclusion and exclusion criteria, demographics, symptoms of PTSD and depression, and the Web Screening Questionnaire for common mental disorders (Donker et al., 2009) to assess for comorbidities. Written information about the study will be given before the screening, including objectives, benefits, risks, and requirements imposed by the study. The participants will be encouraged to download the study information and consent form to keep for future reference. Applicants will undergo a clinical psychiatric assessment interview, which includes the CAPS-5, to determine their eligibility for the study. If the inclusion criteria are met, the applicant will be invited to take part in the research (please see Table 1 for the specific inclusion and exclusion criteria). Decisions on inclusion will be made by the assessor and the principal investigator (Maria Bragesjö) together.

### Sample size {14}

A priori power analysis determined the sample size for comparing HOPE with an active control at the primary endpoint (1 month). Based on prior internet-delivered CBT work showing a between-group effect of Cohen’s *d* = 0.38 [8], we powered the trial to detect a conservative effect of *d* = 0.35 with 80% power at a two-sided *α* = 0.05 under 1:1 randomization. This yields *n* = 129 per group at the primary endpoint. To accommodate 10% data attrition (missing primary outcome), consistent with retention in other therapist-guided digital trials [11], we inflated the sample to *N* = 286 (143 per arm), expected to retain 129 per arm for analysis.

### Recruitment {15}

The study will recruit participants from a psychiatric outpatient clinic in Stockholm. Mental health professionals working at the clinic will have access to an information sheet of the study, which they can present and discuss with patients. Self-referrals are available through a dedicated website, and advertisements will be placed on social media with information about the study.

## Assignment of interventions: allocation

### Sequence generation {16a}

After eligibility confirmation and baseline assessment, participants will be randomized 1:1 to HOPE or the active control, stratified by self-rated complex PTSD (yes/no) and gender. The sequence is computer-generated with variable block sizes and programmed by an external unit (Karolinska Trial Alliance) via a secure, audited, web-based system independent of the clinical team. The sequence is stored server-side and inaccessible to site staff. Allocation is released only after baseline completion through role-based login, ensuring concealment; staff cannot foresee upcoming assignments. On randomization, the system issues a tamper-evident allocation certificate (participant ID, assigned arm, personnel ID, timestamp) and maintains a full audit trail.

### Concealment mechanism {16b}

The allocation sequence in the digital system is concealed from all study personnel.

### Implementation {16c}

The local project leader will assign participants to interventions.

## Assignment of interventions: Blinding

### Who will be blinded {17a}

Group allocation will be masked to the assessors up to completion of the 12-month follow-up. Participants will receive explicit instructions not to discuss their treatment allocation with the assessor. Following each assessment, the assessors will be requested to guess the participants’ allocation, and these responses will be compared to chance at the study’s conclusion to assess the integrity of blinding.

If participants inadvertently disclose the treatment allocation during the assessment, the assessment will stop, and another blind assessor will reassess the diagnosis. The participant will also be assigned another assessor at subsequent follow-ups. The primary outcome assessors and trial statistician will be blinded to group allocation during the whole duration of the trial.

### Procedure for unblinding if needed {17b}

Emergency unblinding will occur when clinically necessary, such as when treatment decisions require knowledge of the intervention or when an unexpected serious adverse event arises. Other instances of unblinding include (1) conducting interim analysis according to the study analysis plan and (2) at the conclusion of the study to assess the intervention’s effects.

## Data collection and management

### Plans for assessment and collection of outcomes {18a}

Participants will complete baseline assessments, weekly measures throughout the treatment period, immediately after treatment completion and at the 1-month, 6-month, and 12-month follow-ups. All data are collected in a secure digital platform (BASS).

### Plans to promote participant retention and complete follow-up {18b}

No reimbursement to participants will be given to complete the assessment. The digital platform sends out automatic reminders if assessments are late.

### Data management {19}

Case report form data will be imputed to a data file. All data will be handled and eventually archived according to Swedish and European legislation. Data will not be made publicly available. Participants will complete online questionnaires via BASS, a password-protected digital platform hosted by the Karolinska Institutes secure computing facilities, which also handle data backup. The platform also uses two-factor authenticating. Participants will complete assessments and interviews with researchers via phone or secure video-conferencing software used at the recruitment site.

### Confidentiality {27}

The data generated in the research project will be managed and stored according to Karolinska Institutet’s and Stockholm County’s Research Data Management Policy. The study personnel involved in the trial are bound by confidentiality principles in their role as employers in the health care system.

### Plans for collection, laboratory evaluation, and storage of biological specimens for genetic or molecular analysis in this trial/future use {33}

Not applicable since the trial does not involve collecting biological specimens for storage.

## Statistical methods

### Statistical methods for primary and secondary outcomes {20a}

The data analyses will be carried out by an independent statistician who is not part of the research group. This statistician will also remain blind to the group allocation throughout the duration of the trial. The intention-to-treat approach to the analysis population will be applied in all cases unless specified otherwise. The significance level for all statistical tests will be *α* = 0.05.

For our primary research questions, we will fit a linear mixed-effects model for repeated measures to evaluate change in CAPS-5 total scores within and between groups over time. Fixed effects will include treatment, time, and the treatment × time interaction; a participant-specific random intercept will be specified. An unstructured within-participant covariance will be used, allowing distinct correlations between all pairs of time points. The model will be estimated by restricted maximum likelihood and will use all available observations, yielding unbiased estimates under missing at random or missing completely at random assumptions. The primary hypothesis will be tested two-sided at *α* = 0.05. No adjustments will be made for multiplicity.

As a complementary check at the primary endpoint, we will fit a linear regression of change in CAPS-5 from baseline to 1-month on randomization arm, adjusting for baseline CAPS-5.

We will report within- and between-group effect size (Cohen’s *d*) with 95% CIs. Effects will be deemed statistically significant at *p* < 0.05. Magnitudes will also be described using conventional benchmarks: small (0.20), medium (0.50), large (≥ 0.80).

Crossover participants continue all scheduled outcome assessments. Post-crossover data are obtained as part of a naturalistic follow-up.

#### Secondary outcomes and analyses

Unless stated otherwise, analyses mirror the primary modelling strategy: continuous outcomes will be analyzed with linear mixed-effects models using all available observations and yielding unbiased estimates under MAR/MCAR. Binary (and ordinal) outcomes will be analyzed with mixed-effects logistic (or cumulative-logit) models; cross-sectional categorical comparisons will use *χ*^2^ or Fisher’s exact tests as appropriate. Effect sizes will be reported as model-based standardized mean differences (Cohen’s *d*) for continuous outcomes and odds ratios for binary outcomes, each with 95% CIs. All secondary analyses are exploratory and *p*-values are nominal (no additional multiplicity adjustment).

#### Health-economic evaluation

A within-trial cost-effectiveness and cost-utility analysis will compare HOPE with the control at each assessment. QALYs will be derived from AQoL-6D using general-population tariffs. Costs will be estimated from a healthcare-provider perspective (intervention costs), a healthcare-system perspective, and a societal perspective using clinic resource use and TIC-P data. Results will be presented as incremental cost-effectiveness ratios (ICERs), with uncertainty characterized via non-parametric bootstrapping, cost-effectiveness planes, and cost-effectiveness acceptability curves across willingness-to-pay thresholds.

The full statistical analysis plan is uploaded on OSF: https://osf.io/dsg9p/.

### Interim analyses {21b}

Interim analysis of the results will be conducted twice during the study by an independent statistician: once when approximately one-third of the participants have completed the 1-month follow-up and again when approximately two-thirds of the participants have completed the 1-month follow-up. The purpose is safety monitoring, with emphasis on adverse events, serious adverse events, and withdrawals due to adverse events (frequency, severity, relatedness, and any between-arm imbalances). Unblinding will occur if required to evaluate a potential safety signal.

### Methods for additional analyses (e.g., subgroup analyses) {20b}

We will explore differences in treatment effects by gender in a subgroup analysis by including an interaction term between treatment arm and gender. Exploratory mediation will evaluate whether change in emotional responding, adherence, post-traumatic cognitions, avoidance, and alliance mediates treatment effects on CAPS-5. Moderation by baseline demographics and clinical history (e.g., PTSD/CPTSD severity, trauma type and timing, childhood adversity, comorbidity, perceived threat, education, employment, credibility/alliance) will be tested via treatment × covariate interactions; for prognostic models of CAPS-5 response, logistic regression will be used with model calibration assessed by Hosmer–Lemeshow and calibration plots.

### Methods in analysis to handle protocol nonadherence and any statistical methods to handle missing data {20c}

All available data will be used in the models. All protocol deviations will be reported.

### Plans to give access to the full protocol, participant-level data, and statistical code {31c}

Consent to share deidentified information for future research is specified within the participant consent forms. Deidentified datasets will be available from the principal investigator upon reasonable request and with formal data transfer agreements for further research. The study protocol and the statistical code used will also be made available.

## Oversight and monitoring

### Composition of the coordinating center and trial steering committee {5d}

The team responsible for recruitment and administrative task of running the trial meets weekly. The group consists of the Principal Investigator, Project Manager, staff responsible for treatment and recruitment activities. No separate Trial Steering Committee is convened for this low-risk study. Independent safety oversight is provided by a statistician/monitor who conducts the prespecified interim safety reviews (see {21b}); the clinical team does not access comparative interim results unless unblinding is required to evaluate a safety signal. Qualitative input from the pilot phase (patients and clinicians) informed participant-facing materials, screening and reminder procedures, questionnaire burden/acceptability, and retention strategies.

### Composition of the data monitoring committee, its role and reporting structure {21a}

The intervention researched in the trial is of low risk, and the Human Research Ethics Committee does not require a Data Monitoring Committee. Independent safety oversight is nevertheless provided by a statistician/monitor who conducts the interim safety reviews described in.

### Adverse event reporting and harms {22}

Participants can report the occurrence of undesirable treatment effects at any point through the study period by alerting the designated therapist on the digital platform or assessor or contacting the study personnel. Weekly measures to capture any unwanted adverse events will be administered, including questions about intensity, duration, and characteristics of the adverse event.

All potential undesirable treatment effects will be categorized by the investigator into three levels: mild, moderate, or severe. An event will be deemed serious if it poses a life-threatening risk, leads to death, causes persistent or significant disability/incapacity, necessitates hospitalization or its prolongation, or is determined to be medically significant by the principal investigator. The Negative Effects Questionnaire (NEQ) will be utilized posttreatment and during follow-ups to systematically gather data on potential adverse effects.

Therapists will be needed to promptly inform the principal investigator if they have concerns that a participant has caused, or is at risk of causing, significant harm to themselves or others or discloses any suicidal ideation. In such cases, a suicide risk assessment will be conducted over the phone to ensure appropriate and immediate support.

All participants in the study will receive active treatments with previous support by clinicians who specialize in the treatment of PTSD. Previous studies that have investigated intensive prolonged exposure have not detected any serious adverse events. The additional risks associated with participating in digital HOPE are hence considered to be small. All participants will be closely monitored during the study, and additional treatment will be provided in case of an unlikely severe deterioration.

Excluded participants will be informed where to seek help in the regular health care system. After the 1-month follow-up, participants will be offered the treatment they were not originally allocated to.

### Frequency and plans for auditing trial conduct {23}

Any potential modifications to the protocol will be fully disclosed on Open Science Framework and Clinicaltrials.org.

### Plans for communicating important protocol amendments to relevant parties (e.g., trial participants, ethical committees) {25}

If needed, amendments to the Swedish Ethical Review Authority will be submitted. Any potential modifications to the protocol will be fully disclosed on Open Science Framework and Clinicaltrials.org.

### Dissemination plans {31a}

Participants in a previous pilot study on HOPE were interviewed about their experiences, and some modifications were made to the treatment protocol accordingly. The obtained findings will have important implications for the treatment of PTSD, and the results hold great importance to a large target group consisting of afflicted individuals and their families, policy makers, clinicians, stakeholders, media, and the public. The results in the project will be communicated to the scientific community by publishing the results in open access, high-impact scientific journals and by presenting our results at several national and international key conferences. The results will further be communicated more widely according to a separate communication plan using state-of-the-art communication tools and help from a communication expert.

We aim to reach both professionals as well as a broad audience of anyone interested in the research project through each of the project’s parties’ websites, social media channels, press releases, local and national newspaper, TV, and radio coverage. Since regular health care is already involved in the project, implementation is expected to be straightforward.

## Discussion

The study is the first trial to evaluate a digital therapist-guided treatment for PTSD (HOPE) in a Swedish psychiatric setting and not exclude patients with severe or complex PTSD and has the potential to impact guidelines on its suitability for that target group. The study is well powered as a superiority trial, and the research group has extensive expertise in trauma and PTSD as well as digital interventions. The therapists and assessors engaged in the trial have undergone comprehensive training and supervision in the treatments being investigated and in the utilization of the clinical gold-standard measure employed to assess PTSD. Several measures to increase the scientific trustworthiness of the study have been taken. The study hypotheses and the data analytic plan were prospectively registered. The chosen outcome measures for the study are considered the gold standard. The results of the trial will be communicated in open access scientific publications, at scientific conferences as well as meetings with representatives from the health care system and patient organizations, and in public media. The trial will be conducted in a regular care setting, which could pose a problem. The therapists delivering the treatment work clinically at the recruitment site and currently have high caseloads, and taking part in the research is an additional responsibility. If a therapist does need to drop out of the study due to other work commitments or for personal reasons, it could take quite some time to recruit new clinicians to replace them and train them in the treatment and the measures used in the study.

In summary, previous work has shown that trauma focused online has the potential to be an effective treatment for PTSD. No study to date has yet compared digital prolonged exposure against an active control condition in a psychiatric setting with patients with moderate to severe PTSD.

## Trial status

Inclusion started on the 4th of September 2023 and is expected to end in August 2026. The last follow-up appointment is expected to take place in October 2027. Data analysis and reporting of results will begin when all data from the primary endpoint have been collected.

## Data Availability

The principal investigator Bragesjö will serve as the data custodian. Deidentified datasets can be obtained from the principal investigator upon reasonable request and under formal data transfer agreements to facilitate additional research.

## Abbreviations

AQoL-6D: Assessing Quality of Life 6 Dimensions
CAPS-5: Clinician-Administered PTSD Scale for DSM-5
CIPE: Condensed internet-delivered prolonged exposure CIPE
CBT: Cognitive behavioral therapy
CEQ: Credibility/Expectancy Questionnaire
CHEERS: Consolidated Health Economic Evaluation Reporting Standards
CONSORT: Consolidated Standards of Reporting Trials
DSM-5: Diagnostic and Statistical Manual of Mental Disorders, fifth edition
EMDR: Eye Movement Desensitization and Reprocessing therapy
HOPE: Huddinge Online Prolonged Exposure
GCP: Good Clinical Practice
ITQ: The International Trauma Questionnaire
NEQ: Negative Effects Questionnaire
OSF: Open Science Framework
RCT: Randomized controlled trial
SUD: Subjective Units of Distress Scale
TIC-P: Treatment Inventory of Costs in Psychiatric Patients
PHQ-9: Patient Health Questionnaire
PTSD: Posttraumatic stress disorder
WAI-SR: Working Alliance Inventory–Short Form Revised
